# Family networks during migration and risk of non-affective psychosis: A population-based cohort study

**DOI:** 10.1016/j.schres.2019.01.044

**Published:** 2019-06

**Authors:** Jennifer Dykxhoorn, Anna-Clara Hollander, Glyn Lewis, Christina Dalman, James B. Kirkbride

**Affiliations:** a Division of Psychiatry, UCL, London, United Kingdom; b Department of Public Health Sciences, Karolinska Institutet, Stockholm, Sweden; c Centre for Epidemiology and Community Medicine, Stockholm County Council, Stockholm, Sweden

**Keywords:** Psychotic disorder, Epidemiology, Social capital, Family network

## Abstract

**Objective:**

The determinants of increased psychosis risk among immigrants remain unclear. Given ethnic density may be protective, we investigated whether the presence of immediate family, or “family networks”, at time of immigration was associated with risk of non-affective psychosis.

**Methods:**

We followed a cohort of migrants (n = 838,717) to Sweden, born 1968–1997, from their 14^th^ birthday, or earliest immigration thereafter, until diagnosis of non-affective psychosis (ICD-9/ICD-10), emigration, death, or 2011. Using record linkage, we measured family network as the presence of adult first-degree relatives immigrating with the cohort participant or already residing in Sweden. We used Cox proportional hazards regression to examine whether risk varied between those migrating with family, migrating to join family, or migrating alone.

**Results:**

Migrating with immediate family was associated with increased psychosis risk amongst males compared to males who did not migrate with family (adjusted Hazard Ratio [aHR]: 1.16, 95% CI: 1.00–1.34). Migrating with family did not increase risk among females (aHR: 0.91, 95% CI: 0.78–1.07); similar observations were observed for males who immigrated to join family (aHR: 1.35, 95% CI: 1.21–1.51). In contrast, females who migrated alone were at increased risk compared to females who did not migrate alone (aHR: 1.31, 95% CI: 1.11–1.54).

**Conclusion:**

Family networks at the time of immigration were associated with differential patterns of non-affective psychotic disorders for males and females. These results suggest sex-specific differences in the perceived role of family networks during the migration process.

## 1 Introduction

There is robust evidence that several migrants groups and their children are at increased risk of psychotic disorders (Bourque et al., 2011; Cantor-Graae and Pedersen, 2013) with exact risk varying by country of origin, ethnicity, and reason for migration (Hollander et al., 2016). While several plausible mechanisms may explain this variation, little empirical evidence exists to support any given hypothesis. One putative explanation is that pre-, during, and post-migratory exposure to social adversity, discrimination, social isolation, and low socioeconomic position may lead to social stress relevant to the onset of psychotic disorder (Adriaanse et al., 2015; Kirkbride et al., 2008a, Kirkbride et al., 2008b; Morgan et al., 2008; Morgan and Hutchinson, 2010). In particular, the availability of social networks may buffer stressors experienced before, during, and after migration (Cohen and Wills, 1985), consistent with observations that risk is modified amongst migrant who live in communities with a higher proportion of people from similar ethnic backgrounds (i.e. the ethnic density hypothesis) (Veling et al., 2008).

Social networks are complex webs of interpersonal relationship comprised of close family and friends and weaker ties to colleagues, neighbours, and community members. Social networks may help buffer stressors experienced before, during, and after migration (Cohen and Wills, 1985), and mitigate risk of subsequent disorder. Social networks are regarded as important sources of social capital, and are at times used synonymously in the literature (Ryan et al., 2008). For example, in his seminal work, Putnam described social capital as ‘social networks and the associated norms of reciprocity and trust’ (Putnam, 2007, Putnam, 2000). Seen through this lens, social networks have been associated with numerous physical and mental health benefits, such as fewer health complaints among children (Eriksson et al., 2012), fewer behavioural and mental health problems among adolescents (McPherson et al., 2014), and lower rates of schizophrenia (Kirkbride et al., 2008a, Kirkbride et al., 2008b), and suicide (Congdon, 2012; Kelly et al., 2009).

Since shared norms, reciprocal ties, and practical assistance are often features of kin-based networks, the presence of family members in one’s immediate social network around the time of immigration may be an important source of social capital (Moskowitz et al., 2012; Rumbaut, 1997; Widmer, 2006), and may therefore mitigate psychosis risk. Social networks have been shown to be particularly important for migrants entering the labour market. Research from Germany showed that 50% of migrants found their jobs through networks, compared with 30% of native-born individuals (Drever and Hoffmeister, 2008). Thus, individuals migrating with family members or to join family already in the host country may have access to more support than individuals who migrate alone. Those migrating alone may lack such resources during the migration and acculturation process. It is also possible that migrating with family members introduce heightened stress for some migrants, counterintuitively increasing subsequent psychosis risk. Despite these possibilities, no epidemiological study to date has inspected whether the presence of immediate family networks during the migration process influences subsequent psychosis risk.

### 1.1 Aims of the study

We sought to investigate whether the availability of family networks around the time of immigration was associated with subsequent risk of non-affective psychosis. We hypothesised that individuals who migrated with first-degree relatives (parents, spouses, siblings), or those who joined family already settled in Sweden would have lower risk of psychotic disorders, while those migrating alone would have higher risk. We expected these effects would be more pronounced in females, given that social support may have a stronger protective effect on mental health in females (Walen and Lachman, 2000). We also hypothesised that family networks would have stronger protective effects for migrants from geographically distant areas, given it might have been more difficult for them to maintain regular social connections with family and friends in their country of origin. Finally, we considered whether migrating with or without dependent children altered psychosis risk amongst migrants, since these experiences may provide additional sources of strain or social support for parents; given the lack of previous research here, we did not set an a priori hypothesis here.

## 2 Methods

### 2.1 Study design and population

We utilized data from Swedish population registers to identify people born between 1968 and 1997 outside of Sweden who later immigrated to, and were living in Sweden with an official residence permit on or after their 14^th^ birthday. Participants were followed from their 14^th^ birthday, or date of first immigration if later, until exit from the cohort due to diagnosis of non-affective psychosis, emigration, death, or 31 December 2011, whichever was sooner. We excluded people born in Sweden, temporary visitors, those without an official residency permit (asylum seekers, undocumented migrants), and people diagnosed with a non-affective in Sweden before their 14^th^ birthday.

### 2.2 Outcome

We linked immigrants to the National Patient Register [NPR] to ascertain diagnoses of non-affective psychotic disorder according to the International Classification of Diseases [ICD], versions 9 (295, 297, 298) or ICD-10 (F20-29). The NPR included both in- (1983–2011) and out-patient records (2001–2011).

### 2.3 Exposure

**Fig. 1:**
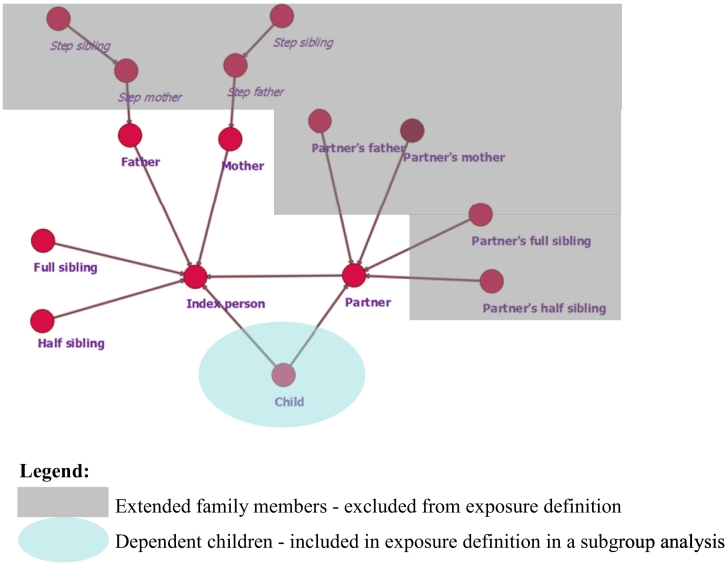
Family members included in family network exposures. **Legend:** Extended family members - excluded from exposure definition Dependent children - included in exposure definition in a subgroup analysis.

We linked migrants with their first-degree relatives living in Sweden (biological parents, adoptive parents, siblings and half-siblings, partners, and children) using the Multigenerational Register. Step-families and in-laws were excluded from analysis. Dependent children (less than 18 years old) at the time of immigration were excluded from our primary exposure, but were considered in a secondary analysis (see below and Fig. 1).

We then determined whether participants immigrated with these family members, joined family members already in Sweden, or migrated alone. Statistics Sweden maintains the immigration and emigration register (STATIV) annually, from which we obtained information on date of first immigration for each participant and their first degree relatives, via linkage to the Multigenerational Register. While a small number of refugees receive their residency permits immediately upon arrival to Sweden, most migrants experience a delay between arrival in Sweden and entry into the migration register. For this reason, we defined any individual who immigrated in the same calendar year as one or more family member(s) as having migrated with family. Any migrants who had one or more family member(s) in Sweden prior to the year of their migration were defined as having joined family upon migration. Finally, lone migrants were individuals who neither migrated with, nor joined family. While nearly 75% of immigration is reported to the Swedish Tax Authority within 10 days, and an additional 20% within 30 days, Statistics Sweden utilizes the population at the end of each calendar year as the baseline population register for research (Ludvigsson et al., 2016).

As a secondary exposure, we considered whether migration with or without dependent children (under age 18) altered later psychosis risk. This analysis was restricted to migrants with dependent children at the time of immigration, as identified through the Swedish registers. Migrants who immigrated in the same calendar year as one or more of their dependent children were considered as having migrated with children. They were compared with migrants who had dependent children at the time of immigration, but who migrated before or after their children.

### 2.4 Confounders

We considered sex, age, time period, age-at-migration, and region of origin as *a priori* confounders. We modelled calendar time as a time-varying covariate to account for possible period effects over the follow-up period (1982–1991, 1992–2001, 2002–2011). We also modelled age as a time-varying covariate, splitting age into the following age bands: 14–18, 19–23, 24–28, 29–33, 34–38, 39–43 years. Age-at-migration was categorized into five groups: infancy (0–2 years), early childhood (3–6 years), middle childhood (7–12 years), adolescence (13–18 years), early adulthood (19–29 years), and adulthood (30 years or older), consistent with previous research (Dykxhoorn et al., 2018). Region of origin obtained from the Total Population Register and grouped into six regions: Europe, Asia + Oceania, Middle East + North Africa, sub-Saharan Africa, North America, and South America. In a sensitivity analysis, we adjusted for refugee status, based on the Swedish Migration Agency's definition of refugee status.

### 2.5 Statistical analysis

We first presented descriptive statistics for the sample. Then, using Cox proportional hazard regression, we investigated whether the incidence of non-affective psychosis amongst migrants varied by each of our family network exposures, in univariable and multivariable models. We reported unadjusted (uHR) and adjusted hazard ratios (aHR), with 95% confidence intervals (95% CI), adjusting for *a priori* confounders and mutual adjustment for the other family network variables in each analysis. Interactions were modelled via likelihood ratio test (LRT), comparing model fit with and without the relevant interaction term at a p-value threshold of p<0.05.Given strong evidence of effect modification by sex, all results were presented stratified by sex. We also tested the interaction between family network exposures and psychosis risk by region.

We tested the proportional hazards assumption using Schoenfeld residual plots to assess departure from proportionality. We conducted a sensitivity analysis using a washout period to exclude those diagnosed within two years of immigration, who may have been prevalent cases. All modelling was conducted in Stata 12.

## 3 Results

### 3.1 Sample characteristics

Among 838,717 migrants included in this cohort (Table 1), we identified 6,016 incident cases of non-affective psychosis during 6,691,485 person-years of follow-up (crude incidence: 89.9 per 100,000 person-years; 95%CI: 87.7–92.2). There were similar numbers of males (50.5%) and females (49.5%) in the cohort. Migrants predominantly originated from Europe (39.0%), the Middle East + North Africa (23.2%), and Asia + Oceania (20.9%). Among migrants, 29.5% immigrated with family members, 9.6% joined family members, although the majority (65.9%) were lone migrants; 11.2% (N = 93,741) had a record of dependent children in the Swedish registers alive at the time of participant immigration. Of this group, 30.1% immigrated to Sweden with at least one dependent child, while the remainder immigrated before or after their dependent children. Table 1 shows additional cohort characteristics.

**Table 1:** Cohort characteristics.

	N	(%)^1^	Cases	(%)^2^	Person-years
Cohort	838,717	100.0	6,016	0.7	6,691,485
Sex					
Male	423,788	50.5	3,584	0.9	3,264,893
Female	414,929	49.5	2,432	0.6	3,426,592
Decade of birth					
1968–1977	352,489	42.0	3,493	1.0	3,764,578
1978–1987	338,754	40.4	2,135	0.6	2,347,931
1988–1997	147,474	17.6	388	0.3	578,976
Region of origin					
Europe	355,404	42.4	2,031	0.6	2,738,818
Asia + Oceania	146,860	17.5	800	0.5	1,071,073
Middle East + North Africa	194,644	23.2	1,657	0.9	1,699,860
Sub-Saharan Africa	76,749	9.2	985	1.3	534,009
North America	24,628	2.9	155	0.6	177,430
South America	40,432	4.8	388	1.0	470,295
Family networks at migration					
Migrating with family	247,303	29.5	2,295	0.9	2,646,642
Not migrating with family	591,414	70.5	3,721	0.6	4,044,844
Migrating to join family	80,497	9.6	853	1.1	827,961
Not migrating to join family	758,220	90.4	5,163	0.7	5,863,524
Migrating alone	552,681	65.9	3,407	0.6	3,723,702
Not migrating alone	286,036	34.1	2,609	0.9	2,967,783
Age-at-migration					
Infancy	50,871	6.1	572	1.1	753,478
Early childhood	63,191	7.5	730	1.2	800,026
Middle childhood	89,117	10.6	903	1.0	1,044,651
Adolescence	119,475	14.2	1,196	1.0	1,194,370
Early adulthood	371,277	44.3	2,049	0.6	2,355,068
Adulthood	144,786	17.3	566	0.4	543,893
Dependent children at migration (n = 93,741)					
Migrating with children	28,203	30.1	228	0.8	238,221
Not migrating with children	65,538	69.9	205	0.3	262,941

1 Column percent.

2 Row percent.

### 3.2 Family network and risk of non-affective psychotic disorders

**Fig. 2:**
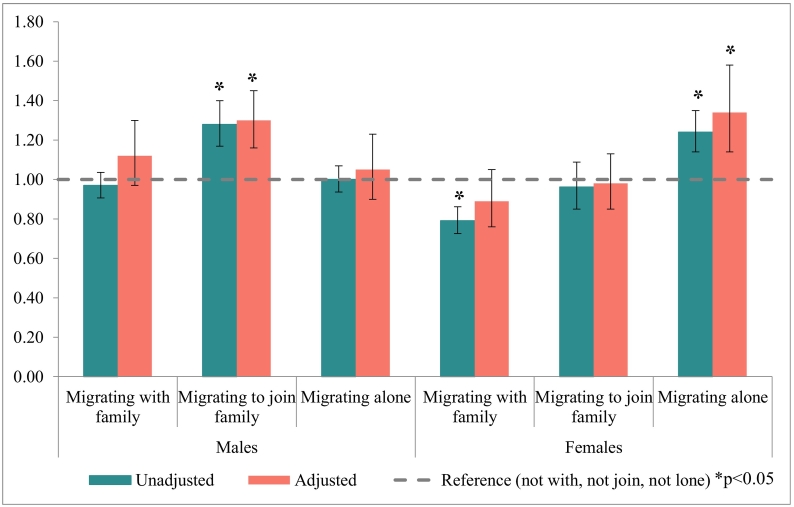
Unadjusted and adjusted hazard ratios of family networks and risk of non-affective psychosis, by sex.

We observed strong effect modification by sex in the relationship between non-affective psychosis risk and family network exposures, so presented stratified results (Supplemental Table 1). For male migrants, those who immigrated to join family were at higher risk of psychosis (aHR: 1.30, 95% CI: 1.16–1.45) compared with those who did not join family members after multivariable adjustment (Table 2, Fig. 2). There was no evidence that migrating with family, or migrating alone altered risk for males in univariable or multivariable models (Table 2).

**Table 2:** Unadjusted and adjusted hazard ratios of family networks and risk of non-affective psychosis, by sex.

	N	(%)	Cases	(%)	Unadjusted			Adjusted^1^		
					HR	95% CI		HR	95% CI	
Males										
Not migrating with family	294,075	40.0	2,069	0.7	1.00			1.00		
Migrating with family	126,129	30.0	1,515	1.2	0.95	0.89	1.01	1.12	0.97	1.30
Not migrating to join family	381,515	90.8	3,016	0.8	1.00			1.00		
Migrating to join family	38,689	9.3	568	1.5	**1.26**	**1.15**	**1.38**	**1.30**	**1.16**	**1.45**
Not migrating alone	144,005	34.3	1,710	1.2	1.00			1.00		
Migrating alone	276,199	65.7	1,874	0.7	1.02	0.95	1.09	1.05	0.90	1.23
Females										
Not migrating with family	293,618	71.2	1,652	0.6	1.00			1.00		
Migrating with family	118,879	28.8	780	0.7	**0.77**	**0.70**	**0.84**	0.89	0.76	1.05
Not migrating to join family	371,542	90.1	2,147	0.6	1.00			1.00		
Migrating to join family	40,955	9.9	285	0.7	0.94	0.83	1.07	0.98	0.85	1.13
Not migrating alone	139,422	33.8	899	0.6	1.00			1.00		
Migrating alone	273,075	66.2	1,533	0.6	**1.28**	**1.18**	**1.39**	**1.34**	**1.14**	**1.58**

HR: Hazard ratio; 95%CI: 95% confidence interval.

Significant p-Values (p<0.05) are in bold.

1 Adjusted for age, time period, age-at-migration, and other family network measures (migrating with family, migrating to join family, migrating alone).

For female migrants (Table 2, Fig. 2), unadjusted estimates suggested that immigrating with family was associated with a reduced incidence of non-affective psychotic disorders compared with those who migrated without family (uHR: 0.77, 95% CI: 0.70–0.84), but this effect disappeared following adjustment for confounders (aHR: 0.89, 95%CI: 0.76–1.05). While there was also no evidence that migrating to join family affected psychosis risk (Table 2), when compared to female migrants with some form of family capital at immigration, those who migrated alone were at increased psychosis risk (aHR: 1.34, 95% CI: 1.14-1.58).

### 3.3 Family network, region of origin, and risk of non-affective psychotic disorders

Risk of psychotic disorders varied according to region of origin and sex (Supplemental Table 1). For males, for example, those migrating with family from Europe (aHR: 1.27, 95% CI: 1.07–1.52) and sub-Saharan Africa (aHR: 1.30, 95%CI: 1.06–1.60) were at elevated risk compared with those migrating without family, while such risks were lower for males from Middle East + North Africa who migrated with family, although the effect size was modest (aHR: 0.82, 95% CI: 0.68–0.98). The overall increased risk of non-affective psychotic disorders amongst males who migrated to join family was independently observed for those from Europe, sub-Saharan Africa, and South America (Table 3). Only males from the Middle East + North Africa were at increased risk when they migrated alone (aHR: 1.49, 95% CI: 1.23–1.80).

**Table 3:** Adjusted hazard ratios of family networks and risk of non-affective psychosis, by region of origin and sex.

		N	(%)	Cases	(%)	Males^1^			Females^2^		
						Adj. HR	95% CI		Adj. HR	95% CI	
Migrating with family											
Europe	Not migrating with family	249,011	70.1	1,142	0.5	1.00			1.00		
	Migrating with family	106,393	29.9	889	0.8	**1.27**	**1.07**	**1.52**	1.00	0.83	1.21
Asia + Oceania	Not migrating with family	122,000	83.1	636	0.5	1.00			1.00		
	Migrating with family	24,860	16.9	164	0.7	1.26	0.97	1.65	0.91	0.67	1.24
Middle East + North Africa	Not migrating with family	116,570	59.9	945	0.8	1.00			1.00		
	Migrating with family	78,074	40.1	712	0.9	**0.82**	**0.68**	**0.98**	0.82	0.66	1.02
Sub-Saharan Africa	Not migrating with family	57,927	75.5	659	1.1	1.00			1.00		
	Migrating with family	18,822	24.5	326	1.7	**1.30**	**1.06**	**1.60**	0.89	0.69	1.16
North America	Not migrating with family	19,200	78.0	110	0.6	1.00			1.00		
	Migrating with family	5,428	22.0	45	0.8	1.21	0.77	1.89	0.70	0.37	1.31
South America	Not migrating with family	26,706	66.1	229	0.9	1.00			1.00		
	Migrating with family	13,726	34.0	159	1.2	1.02	0.77	1.36	**0.68**	**0.48**	**0.98**

Migrating to join family											
Europe	Not joining family	328,075	92.3	1,742	0.5	1.00			1.00		
	Migrating to join family	27,329	7.7	289	1.1	**1.59**	**1.33**	**1.89**	1.06	0.85	1.32
Asia + Oceania	Not joining family	137,149	93.4	745	0.5	1.00			1.00		
	Migrating to join family	9,711	6.6	55	0.6	1.19	0.84	1.70	0.73	0.45	1.16
Middle East + North Africa	Not joining family	171,437	88.1	1,448	0.8	1.00			1.00		
	Migrating to join family	23,207	11.9	209	0.9	1.00	0.83	1.21	1.04	0.81	1.34
Sub-Saharan Africa	Not joining family	63,816	83.2	783	1.2	1.00			1.00		
	Migrating to join family	12,933	16.9	202	1.6	**1.27**	**1.04**	**1.56**	0.99	0.75	1.31
North America	Not joining family	22,866	92.9	135	0.6	1.00			1.00		
	Migrating to join family	1,762	7.2	20	1.1	1.69	0.95	3.00	1.13	0.48	2.63
South America	Not joining family	34,877	86.3	310	0.9	1.00			1.00		
	Migrating to join family	5,555	13.7	78	1.4	**1.61**	**1.18**	**2.18**	0.97	0.61	1.53

Migrating alone											
Europe	Not migrating alone	120,261	33.8	1,022	0.9	1.00			1.00		
	Migrating alone	235,143	66.2	1,009	0.4	0.87	0.72	1.04	**1.22**	**1.00**	**1.48**^2^
Asia + Oceania	Not migrating alone	30,720	20.9	183	0.6	1.00			1.00		
	Migrating alone	116,140	79.1	617	0.5	1.02	0.77	1.33	**1.47**	**1.09**	**1.98**
Middle East + North Africa	Not migrating alone	87,733	45.1	769	0.9	1.00			1.00		
	Migrating alone	106,911	54.9	888	0.8	**1.49**	**1.23**	**1.80**	**1.43**	**1.14**	**1.79**
Sub-Saharan Africa	Not migrating alone	24,833	32.4	399	1.6	1.00			1.00		
	Migrating alone	51,916	67.6	586	1.1	0.94	0.75	1.17	**1.32**	**1.02**	**1.72**
North America	Not migrating alone	6,313	25.6	56	0.9	1.00			1.00		
	Migrating alone	18,315	74.4	99	0.5	0.90	0.58	1.39	1.50	0.84	2.68
South America	Not migrating alone	16,176	40.0	180	1.1	1.00			1.00		
	Migrating alone	24,256	60.0	208	0.9	1.22	0.91	1.64	**1.74**	**1.22**	**2.50**

HR: Hazard ratio; 95% CI: 95% confidence interval.

Significant p-Values (p<0.05) are in bold.

1 Adjusted for age, time period, age-at-migration, and other family network measures (migrating with family, migrating to join family, migrating alone).

2 p = 0.048.

The overall raised rates of non-affective psychoses amongst females who migrated alone were independently observed amongst those from Asia + Oceania, Middle East + North Africa, and South America, with weaker trends in this direction for female migrants from Europe, sub-Saharan Africa, and North America (Table 3). No region-specific effects of migrating with or to join family were observed for females, except for a reduced risk of non-affective psychosis for those from South America who migrated with family (aHR: 0.68, 95% CI: 048–0.98).

### 3.4 Timing of migration in relation to dependent children and risk of non-affective psychotic disorders

When restricting the sample to those with dependent children at the time of migration, male migrants were most likely to migrate before their dependent children (89.7%) while the remaining 10.3% migrated with or after their dependents. 17.3% of males migrated with dependents, while most did not migrate at the same time as their children (82.8%). A small minority (1.8%) migrated after their dependent(s) and 98.2% migrated either before or with their children. Males who immigrated with their dependent children had an increased risk of non-affective psychosis compared with those who did not (aHR: 1.62, 95% CI: 1.11–2.36). Most female migrants also immigrated before their dependent children (76.6%) while 23.3% did not migrate before a dependent child. 36.2% of females migrated at the same time as their dependent(s). For female migrants, we observed no differences in risk amongst those who immigrated with their children compared with those who did not immigrate with their children (aHR: 1.17, 95% CI: 0.91–1.52).

### 3.5 Sensitivity analyses

In a sensitivity analysis, excluding potentially prevalent cases of non-affective disorder diagnosed within two years of immigration, our findings showed similar trends (Supplemental Tables 2 & 3). We also controlled for refugee status was conducted on 74.8% of our cohort with information on refugee status, which did not lead to substantially altered results (data available from authors). We tested the proportional hazards assumption from these models, but found no evidence of departure from proportionality (Supplemental Table 4 & Supplemental Figure 1).

## 4 Discussion

### 4.1 Principal findings

In the first study to investigate whether family networks during migration influenced non-affective psychosis risk, we found differential effects for males and females according to the presence or absence of first-degree relatives migrating with or already living in Sweden at the time of migration. Our results suggested that lone female migrants were at greater risk of developing psychosis than those with some family network at the time of migration, measured via the presence of one or more adult first-degree relatives, consistent with our hypothesis. This result extended to females from most regions of origin. In contrast, our results suggested that male migrants who moved with family or to join family were at higher risk of psychosis than males without corresponding markers of family networks. These results were most consistently observed in males migrating from Europe and sub-Saharan Africa, and were impervious to adjustment for age, time period, and region of origin.

### 4.2 Strengths & limitations

This study used large population-based registers with nearly complete coverage. We utilized a novel methodology for estimating measures of family networks amongst immigrants to Sweden, leveraging familial linkages in the multigenerational register. This was based on some assumptions: (i) linkages were restricted to first-degree relatives, and so may have underestimated family capital available from broader family networks, as well as social capital conferred via friendship, kin, and peer groups; (ii) date of immigration was taken from the STATIV (migration) register. Our findings do not therefore generalize to shorter term migrants (typically people visiting Sweden for less than one year), asylum seekers, or undocumented migrants without official residency in Sweden; (iii) we assumed that first-degree relatives who migrated together were given the same dates of immigration in the STATIV database, though to allow for possible variation introduced by administrative delays in processing immigration records (i.e. particularly for refugees seeking asylum in Sweden) we chose a sensitive definition of “migrating with family” to capture all immigration entries within the same calendar year; (iv) we did not have data on the frequency, quality, or strength of family ties; not all relationships will be strong or reciprocal (Widmer, 2006), and we did not have subjective information about who our cohort participants perceived as source(s) of social support. We chose to focus on first-degree relatives, as these are the relationships most likely to be characterized by strong ties, however, we acknowledge that other family members may be important sources of social support, which were not estimated in our study. Indeed, migrants’ networks may be comprised of wider kin relationships or networks with members of a diaspora community who share similar ethnic backgrounds or migratory experiences. We did not adjust for neighbourhood characteristics upon arrival to Sweden as these exposures follow the immigration event and may therefore be on the causal pathway. Furthermore, the decision to immigrate with, or to join family is very unlikely to have been affected by post-immigration neighbourhood characteristics in Sweden, and therefore would be unlikely to have confounded the exposure-outcome associations in this study. Despite these limitations, our novel measure of family networks was able to directly measure the presence of likely sources of social networks during migration and settlement.

Our outcome measure relied on register-based clinical diagnoses of non-affective psychosis, known to be valid for research purposes (Dalman et al., 2002; Ekholm et al., 2009; Ludvigsson et al., 2011). Nevertheless, the registers only included individuals who sought care and received a diagnosis. In theory, differential ascertainment bias could have explained the observed increased risk in males who immigrated to join family, if mental help-seeking behaviours were influenced by family support and knowledge of psychiatric care in Sweden. In general, however, psychotic disorders are serious mental illnesses which usually lead to hospital contact.

Analyses restricted to migrants with dependent children only included participants whose children had immigrated to Sweden. Some immigrants excluded from these analyses may have, in fact, had dependent children in their country of origin, but who had not immigrated to Sweden before the end of our follow-up period. This may have biased our results by misestimating true psychosis risk in those who actually immigrated to Sweden without their dependent children; most plausibly we would have underestimated risk in this group if we failed to include the full sample of people exposed to stresses associated with leaving family behind when migrating to Sweden. If this was the case, the observed excess risk amongst males who immigrated with dependent children, relative to those who did not, may have been overestimated.

Finally, we could not control for some putative confounders in any of our analyses, including educational attainment or socioeconomic position, or prior trauma exposure which were not available prior to arrival in Sweden. We adjusted for refugee status in a secondary analysis but this did not alter our results.

### 4.3 Meaning of findings

Family network appeared to be protective for female migrants, but not for males. This aligns with previous research which has found that family strain predicted psychological and physical health problems in females but not in males (Walen and Lachman, 2000). Further, this research found that social support buffered the effects of stressful situations to a greater extent for females than males (Walen and Lachman, 2000). These differences may arise due to the gendered roles and expectations surrounding immigration experiences for males and females. Recent research on post-migration difficulties in Sweden suggests that males report significantly higher post-migration stress than females, particularly regarding financial, healthcare, and discrimination issues (Steel et al., 2017). One possibility is that male and female migrants perceive the experience of migrating with or without family differently. Males, who migrate with or to join family, were at higher risk of psychosis in our study, and it is possible that this group perceive the experience of caring for family as a source of post-migratory stress. This effect was particularly pronounced amongst males who migrated with dependent children, who may perceive or experience additional pressures arising from simultaneously navigating childcare and educational systems in addition to securing employment, housing, and healthcare. By contrast, females who migrated alone were at greater psychosis risk, raising the possibility that families provide an important source of social support for this group, potentially buffering acculturative stressors or reducing social isolation (Anjara et al., 2017). This is consistent with evidence suggesting that female migrants entering post-migratory labour markets experience more structural barriers to participation in securing employment (Llácer et al., 2007; Milewski et al., 2018; Riaño and Baghdadi, 2007), a task potentially made more stressful without additional family support (Riaño and Baghdadi, 2007). Migrant women are also more likely to be involved in precarious or exploitative jobs with little opportunity for career advancement (Llácer et al., 2007; Vissandjée et al., 2011). Further, gender normative roles could exert differential pressure on men and women during migration. While to some extent, females who migrant may resist normative gender roles by achieving financial independence and autonomy, some report a high sense of family obligation and the expectation to sacrifice her needs in order to send remittances to family in the country of origin.

It is also possible that the elevated psychosis risk we observed for males who migrated to join family could have, paradoxically, been partially explained by the healthy immigrant effect, if males that followed family who had initially immigrated to Sweden to establish work and housing were more vulnerable to psychosis than the index family migrant. For example, if those who joined family in Sweden differed in characteristics which may also confer increased psychosis risk (such as lower SES, education, differences in resilience, or previous mental health difficulties), they may have been more vulnerable to later psychosis than the initial family member. If this were the case, we may have expected these results to disappear in sensitivity analyses, which excluded putatively prevalent cases of psychosis in migrants who presented with psychotic disorder within two years of arrival to Sweden. However, our results were impervious to such selection effects. Alternately, elevated risk observed amongst male migrants who joined family members could also result from longer exposures to social adversities in their country of origin than family members who first emigrated. “Push factors” such as political instability, lack of economic opportunities, or famine can motivate an individual to migrate (Boswell, 2002; Parkins, 2015).

Sex, ethnicity, and social class are all important social determinants of health, and the intersection of these identities (for example female migrants from minority ethnic groups) experience additional dimensions of risk before, during, and after migration. Female migrants may be more likely to experience trauma prior to migration, violence during the migration journey, and additional barriers to employment, education, or income in the host country (Llácer et al., 2007). This analysis revealed differences in the patterns of risk for male and female migrants, however, there are still many knowledge gaps in how sex and gender interact with the determinants of mental health to result in increased risk of psychotic disorders. There is some evidence that the appraisal of social support varies by sex, where males and females may have different expectations of social support, which could be explained by differing socialization experiences or social roles (Matud et al., 2003). Further, perception of social support has been shown to depend on an individual’s country of origin, culture, and ethnicity (Stewart et al., 2010). Indeed, the presence of a family member in Sweden may not directly correspond to increased social contact, stronger family networks, or increased support. Various factors may influence these differences, including differential pathways to care and gendered patterns of exposure to social stressors and sources of support within and outside of one’s immediate family. Clearly, replication of our findings is a necessary perquisite to further discussion of these findings, and will help establish the generalizability of this research in other contexts.

We had theorized that geographic distance may explain some regional variation in risk by levels of family networks, as migrants from distant countries may have fewer opportunities to return to home country or maintain kinship and friendship ties. However, our results did not reveal a simple relationship between geographic distance and psychosis risk. This may be because migrant groups from different regions are likely to vary in visible minority status and migrant type (i.e. voluntary labour migration, family migration, asylum seeking, or refugee). Consistent with the overall trends, lone female migrants were consistently at elevated risk, regardless of region of origin, although some estimates were non-significant, possibly due to low power. Similarly, males from both proximal (i.e. Europe) and distal (i.e. Asia + Oceania, Africa, and South America) regions who migrated with or to join family were at elevated risk, with no consistent pattern by region of origin.

Availability of family networks during immigration to Sweden, as measured via the presence or absence of first-degree relatives, had differential effects on psychosis risk for males and females. In our study, the presence of an immediate family network was protective for female migrants but increased risk for males. It is possible that gendered experiences encountered in the context of immigration may contribute to some of this heterogeneity, and moreover, may underpin some of the excess rates of psychotic disorders amongst immigrant groups (Bourque et al., 2011; Cantor-Graae and Pedersen, 2013). More detailed measures of individual perceptions of family capital will help shed light on the contribution of familial and social networks on the development of non-affective psychosis among migrants.

## Conflict of interest

All authors declare that they have no conflicts of interest to disclose.

## Contributors

All authors contributed to the design of the study and analytic plan. JD conducted the statistical analysis and wrote the first draft of the manuscript. All authors contributed to and have approved the final manuscript

## Funding body agreements and policies

This work was supported by a Sir Henry Dale Fellowship jointly funded by the Wellcome Trust and the Royal Society (grant number: 101272/Z/13/Z to JBK), by Mental Health Research UK (to JD) and by a UCL Overseas Research Scholarship (to JD). JD, GL, JK are also supported by the National Institute for Health Research, University College London Hospital, Biomedical Research Centre.

## Appendix A Supplementary data

Supplementary material Image 3
