# Supplementary material for: Family networks during migration and risk of non-affective psychosis: A population-based cohort study
Source: Schizophr Res. 2019 Jun;208:268–75. doi: 10.1016/j.schres.2019.01.044 (PMC6551364; doi:10.1016/j.schres.2019.01.044)
Supplement: Supplementary file 1 — Supplementary material [file mmc1.doc]

**Supplementary material: Family capital during migration and risk of non-affective psychosis: A population-based cohort study**

Supplemental table 1: Likelihood ratio tests for effect modification by sex and sex & region

|  | **p-value** | |
| --- | --- | --- |
| **Sex** |  |  |
| Sex * migrating with | <0.001 | |
| Sex * migrating to join | <0.001 | |
| Sex * migrating alone | <0.001 | |
|  | **p-value for males** | **p-value for females** |
| **Region** |
| Region * migrating with | 0.001 | 0.29 |
| Region * migrating to join | 0.02 | 0.76 |
| Region * migrating alone | <0.001 | 0.31 |

Supplemental table 2: Family networks at migration and risk of non-affective psychosis (2-year washout period), by sex

|  | N | (%) | Cases | (%) | Unadjusted | | | Adjusted^1^ | | |
| --- | --- | --- | --- | --- | --- | --- | --- | --- | --- | --- |
|  |  |  |  |  | HR | 95% CI | | HR | 95% CI | |
| **Males** |  |  |  |  |  |  |  |  |  |  |
| Not migrating with family | 295,661 | 30.1 | 1,586 | 0.5 | 1.00 |  |  | 1.00 |  |  |
| Migrating with family | 127,539 | 69.9 | 1,410 | 1.1 | 1.04 | 0.97 | 1.12 | 1.07 | 0.91 | 1.25 |
| Not migrating to join family | 383,981 | 90.7 | 2,466 | 0.6 | 1.00 |  |  | 1.00 |  |  |
| Migrating to join family | 39,219 | 9.2 | 530 | 1.4 | **1.35** | **1.23** | **1.48** | 1.32 | 1.18 | 1.48 |
| Not migrating alone | 145,587 | 34.4 | 1,582 | 1.1 | 1.00 |  |  | 1.00 |  |  |
| Migrating alone | 277,613 | 65.6 | 1,414 | 0.5 | 0.93 | 0.86 | 1.00 | 0.99 | 0.83 | 1.17 |
| **Females** |  |  |  |  |  |  |  |  |  |  |
| Not migrating with family | 294,880 | 71.2 | 1,262 | 0.4 | 1.00 |  |  | 1.00 |  |  |
| Migrating with family | 119,570 | 28.9 | 691 | 0.6 | **0.83** | **0.76** | **0.91** | **0.84** | **0.71** | **0.99^2^** |
| Not migrating to join family | 373,246 | 90.1 | 1,704 | 0.5 | 1.00 |  |  | 1.00 |  |  |
| Migrating to join family | 41,204 | 9.9 | 249 | 0.6 | 1.00 | 0.88 | 1.15 | 0.98 | 0.84 | 1.14 |
| Not migrating alone | 140,209 | 33.8 | 787 | 0.6 | 1.00 |  |  | 1.00 |  |  |
| Migrating alone | 274,241 | 66.2 | 1,166 | 0.4 | **1.18** | **1.08** | **1.29** | **1.28** | **1.07** | **1.53** |

HR: Hazard ratio; 95%CI: 95% confidence interval

^1^Adjusted for age, time period, and other family network measures (migrating with family, migrating to join family, migrating alone)

^2^ p=0.043

Supplemental table 3: Unadjusted and adjusted hazard ratios of family networks and risk of non-affective psychosis, by region and sex (2-year washout period)

|  |  | N | (%) | Cases | (%) | Males^1^ | |  | Females^1^ | |  |
| --- | --- | --- | --- | --- | --- | --- | --- | --- | --- | --- | --- |
|  |  |  |  |  |  | Adj. HR | 95% CI | | Adj. HR | 95% CI | |
| **Migrating with family** | |  |  |  |  |  |  |  |  |  |  |
| Europe | Not migrating with family | 248,753 | 70.1 | 884 | 0.4 | 1.00 |  |  | 1.00 |  |  |
|  | Migrating with family | 106,325 | 29.9 | 821 | 0.8 | 1.18 | 0.98 | 1.42 | 0.95 | 0.78 | 1.17 |
| Asia + Oceania | Not migrating with | 121,869 | 83.1 | 505 | 0.4 | 1.00 |  |  | 1.00 |  |  |
|  | Migrating with family | 24,830 | 16.9 | 134 | 0.5 | 1.10 | 0.82 | 1.46 | 0.77 | 0.55 | 1.08 |
| Middle East + North Africa | Not migrating with | 116,321 | 59.9 | 696 | 0.6 | 1.00 |  |  | 1.00 |  |  |
|  | Migrating with family | 78,020 | 40.2 | 658 | 0.8 | 0.85 | 0.70 | 1.03 | **0.79** | **0.62** | **1.00^2^** |
| Sub-Saharan Africa | Not migrating with | 57,739 | 75.5 | 471 | 0.8 | 1.00 |  |  | 1.00 |  |  |
|  | Migrating with family | 18,790 | 24.6 | 264 | 1.6 | **1.26** | **1.01** | **1.58** | 0.92 | 0.68 | 1.21 |
| North America | Not migrating with | 19,173 | 78.0 | 83 | 0.4 | 1.00 |  |  | 1.00 |  |  |
|  | Migrating with family | 5,423 | 22.1 | 40 | 0.7 | 1.14 | 0.70 | 1.85 | 0.64 | 0.32 | 1.27 |
| South America | Not migrating with family | 26,686 | 66.0 | 209 | 0.8 | 1.00 |  |  | 1.00 |  |  |
|  | Migrating with family | 13,721 | 34.0 | 154 | 1.1 | 0.97 | 0.73 | 1.30 | **0.63** | **0.43** | **0.92** |
|  |  |  |  |  |  |  |  |  |  |  |  |
| **Migrating to join family** | |  |  |  |  |  |  |  |  |  |  |
| Europe | Not joining family | 327,763 | 92.3 | 1,430 | 0.4 | 1.00 |  |  | 1.00 |  |  |
|  | Migrating to join family | 27,315 | 7.7 | 275 | 1.0 | **1.56** | **1.31** | **1.87** | 1.06 | 0.84 | 1.34 |
| Asia + Oceania | Not joining family | 136,991 | 93.4 | 587 | 0.4 | 1.00 |  |  | 1.00 |  |  |
|  | Migrating to join family | 9,708 | 6.6 | 52 | 0.5 | 1.32 | 0.92 | 1.89 | 0.77 | 0.47 | 1.27 |
| Middle East + North Africa | Not joining family | 171,160 | 88.1 | 1,171 | 0.7 | 1.00 |  |  | 1.00 |  |  |
|  | Migrating to join family | 23,181 | 11.9 | 183 | 0.8 | 1.05 | 0.86 | 1.28 | 0.96 | 0.73 | 1.27 |
| Sub-Saharan Africa | Not joining family | 63,626 | 83.1 | 593 | 0.9 | 1.00 |  |  | 1.00 |  |  |
|  | Migrating to join family | 12,903 | 16.9 | 172 | 1.3 | 1.23 | 0.99 | 1.53 | 1.04 | 0.76 | 1.41 |
| North America | Not joining family | 22,835 | 92.8 | 104 | 0.5 | 1.00 |  |  | 1.00 |  |  |
|  | Migrating to join family | 1,761 | 7.2 | 19 | 1.1 | 1.68 | 0.92 | 3.06 | 1.29 | 0.54 | 3.05 |
| South America | Not joining family | 34,852 | 86.3 | 285 | 0.8 | 1.00 |  |  | 1.00 |  |  |
|  | Migrating to join family | 5,555 | 13.7 | 78 | 1.4 | **1.64** | **1.20** | **2.23** | 1.05 | 0.66 | 1.67 |
|  |  |  |  |  |  |  |  |  |  |  |  |
| **Migrating alone** | |  |  |  |  |  |  |  |  |  |  |
| Europe | Not migrating alone | 120,183 | 33.9 | 944 | 0.8 | 1.00 |  |  | 1.00 |  |  |
|  | Migrating alone | 234,895 | 66.2 | 761 | 0.3 | 0.87 | 0.71 | 1.05 | 1.20 | 0.97 | 1.48 |
| Asia + Oceania | Not migrating alone | 30,689 | 20.9 | 152 | 0.5 | 1.00 |  |  | 1.00 |  |  |
|  | Migrating alone | 116,010 | 79.1 | 487 | 0.4 | 1.06 | 0.79 | 1.41 | **1.52** | **1.09** | **2.11** |
| Middle East + North Africa | Not migrating alone | 87,662 | 45.1 | 698 | 0.8 | 1.00 |  |  | 1.00 |  |  |
|  | Migrating alone | 106,679 | 54.9 | 656 | 0.6 | **1.37** | **1.12** | **1.69** | **1.44** | **1.13** | **1.84** |
| Sub-Saharan Africa | Not migrating alone | 24,784 | 32.4 | 350 | 1.4 | 1.00 |  |  | 1.00 |  |  |
|  | Migrating alone | 51,745 | 67.6 | 415 | 0.8 | 0.90 | 0.71 | 1.15 | 1.22 | 0.92 | 1.64 |
| North America | Not migrating alone | 6,307 | 25.6 | 50 | 0.8 | 1.00 |  |  | 1.00 |  |  |
|  | Migrating alone | 18,289 | 74.4 | 73 | 0.4 | 0.92 | 0.58 | 1.48 | 1.48 | 0.78 | 2.78 |
| South America | Not migrating alone | 16,171 | 40.0 | 175 | 1.1 | 1.00 |  |  | 1.00 |  |  |
|  | Migrating alone | 24,236 | 60.0 | 188 | 0.8 | 1.19 | 0.88 | 1.62 | **1.73** | **1.18** | **2.53** |

HR: Hazard ratio; 95%CI: 95% confidence interval

^1^Adjusted for age, time period, and other family network measures (migrating with family, migrating to join family, migrating alone)

^2^ p=0.049

Supplemental table 4: Test of proportional hazards assumption

|  | Chi^2^ | df | p |
| --- | --- | --- | --- |
| **Sex** |  |  |  |
| Female | 19.63 | 16 | 0.24 |
| Male | 44.07 | 16 | <0.001* |
| **Region** |  |  |  |
| Europe | 23.98 | 11 | 0.01* |
| Asia + Oceania | 14.50 | 11 | 0.21 |
| Middle East + North Africa | 12.83 | 11 | 0.30 |
| Sub-Saharan Africa | 12.21 | 11 | 0.35 |
| North America | 4.09 | 11 | 0.97 |
| South America | 6.37 | 11 | 0.85 |

* Global p-value <0.05, suggesting possible departure from proportionality. Assumption further examined in Schoenfeld residual plots (see Supplemental Figure 1).


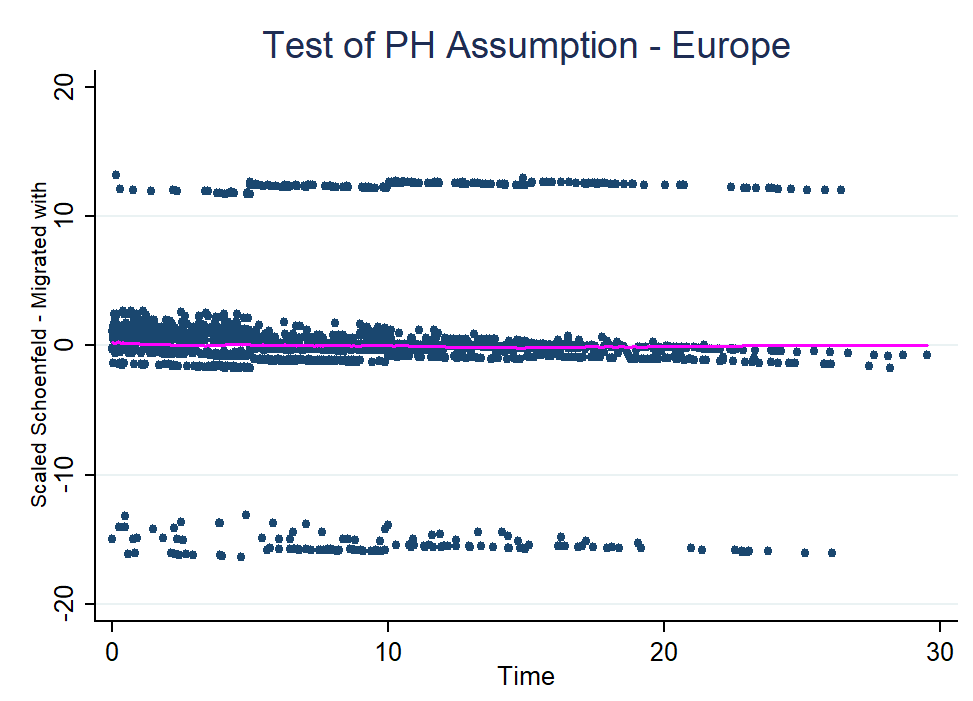

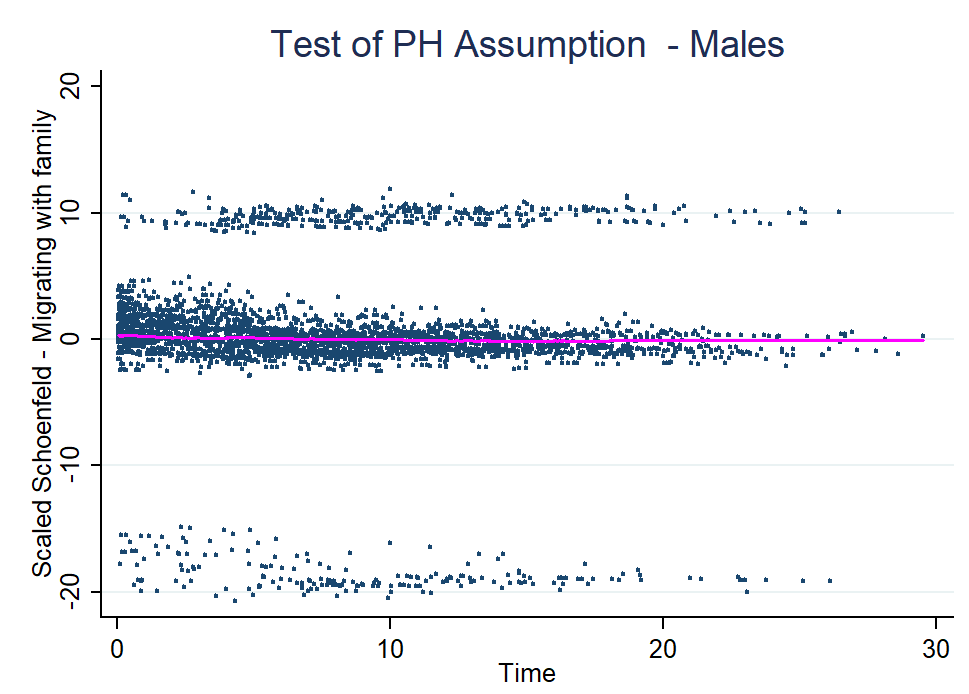


Supplemental figure 1: Schoenfeld residual plots

1A

1B

1A

**Legend**: Figures 1A & 1B show log-log plots of Schoenfeld residuals for males and European migrants, respectively, as the global test had suggested violation of the proportional hazards assumption for these stratum (Supplemental Table 3). Inspection of these plots, fitted with age, sex, and time period suggested little departure from zero slope for the residual line over time, indicating proportional hazards were not violated.
